# Relationship status among lesbian and heterosexual couples 8–10 years after undergoing assisted reproductive treatment in Sweden

**DOI:** 10.48101/ujms.v129.10698

**Published:** 2024-09-05

**Authors:** Konstantinos Chasapis, Gunilla Sydsjö, Agneta Skoog Svanberg, Claudia Lampic, Evangelia Elenis

**Affiliations:** a Department of Women’s and Children’s Health, Uppsala University, Uppsala, Sweden; b Reproduction Center, Women’s Clinic, Uppsala University Hospital, Uppsala, Sweden; c Department of Obstetrics and Gynecology, Linköping University, Linköping, Sweden; d Department of Biomedical and Clinical Sciences, Linköping University, Linköping, Sweden; e Department of Psychology, Umeå University, Umeå, Sweden

**Keywords:** Assisted reproduction, IVF, lesbian relationship, oocyte donation, relationship satisfaction, separation, Sperm donation

## Abstract

**Background:**

Infertility along with fertility treatments has been reported to have a devastating effect on the well-being of the individuals involved as well as their relationship. So far, the studies exploring the impact on the relationship have mainly focused on heterosexual couples facing infertility and undergoing treatment. There is, therefore, a lack of data on the potential role of sexual orientation, gamete origin, as well as treatment success on the risk of separation after fertility treatment. The purpose of this study was, thus, to explore whether sexual orientation, donation treatment, and fertility success affected the relationship well-being and to explore various separation-related aspects.

**Methods:**

We have performed a prospective cohort study of heterosexual and homosexual couples undergoing fertility treatment with autologous and donated gametes in Sweden and followed them for up to 10 years after receiving fertility treatment. In the current follow-up study, 660 individuals have been included.

**Results:**

Almost 39% of lesbian couples participating reported having separated as opposed to 11–17% of heterosexual couples undergoing treatment with own or donated gametes. Neither background factors nor treatment success protected against separation. By using the relationship satisfaction *ENRICH* tool, we were able to demonstrate that dissatisfaction of one of the lesbian spouses or heterosexual spouses undergoing oocyte donation increased significantly the risk of separation 8–10 years after treatment commencement.

**Conclusion:**

The findings can be used by fertility clinics to provide relationship tools to the treated couples in order to help them nurture their relationship and decrease the risk of separation in the long run.

## Introduction

About 10–15% of childbearing-age population in Sweden experience infertility; the vast majority of whom will turn to assisted reproduction technologies (ARTs) to conceive. Globally, most fertility treatments involve the use of couple’s own gametes, with only about 10% utilizing donated gametes (1).

Research indicates that depression and anxiety are common conditions among individuals with fertility problems (2), affecting both their quality of life and the dynamics of their relationship (3), with different impact observed between genders (4). A longitudinal cohort study conducted in Germany, involving 275 couples diagnosed with infertility, revealed a gradual decline in relationship satisfaction over time (5). This decline was particularly pronounced among women and appeared unaffected by the success of ART treatment (5). Additionally, a study performed in China, which included women treated with either In vitro fertilization (IVF) or Intracytoplasmic sperm injection (ICSI), as well as a fertile control group, found that those in the former two groups reported worse psychological status and lower marital quality compared to the fertile group (6). Similarly, a systematic review by Tao et al. demonstrated that the presence of infertility had a more profound negative effect on women’s marital relationship than on men’s (7). In fact, Kjaer et al. in a cohort study from Denmark found that nearly a third of female patients were no longer cohabiting with their partners 12 years after infertility assessment, with a higher likelihood observed among couples who did not achieve parenthood after treatment (8). Regarding men, Tao et al. concluded that the diagnosis of infertility did not have a negative impact on their marriage; on the contrary, men reported a higher level of satisfaction compared to their female partners (7). Similarly, Schanz et al. demonstrated that male psychological well-being and satisfaction with partnership remained unaffected 5 years after infertility diagnosis, regardless of whether they were able to father a child or not (9).

Regarding the origin of gametes (donated or autologous) used in ART and its association with relationship well-being, data are limited. Two studies explored the question and demonstrated equally solid relationships among heterosexual couples independently if treated with autologous or donated gametes, with higher marital satisfaction reported by women receiving treatment with donated oocytes (10, 11). Marital satisfaction has also been explored in relation to sexual orientation. Both lesbian and heterosexual couples reported experiencing a decline in relationship satisfaction after completing their fertility treatment, albeit with slight differences noted; lesbian couples were more satisfied with their relationship at the beginning of treatment (12, 13).

Regarding the presence of children after ART and the risk of separation, the findings are inconclusive. A longitudinal study performed in Great Britain examined the relationship stability and relationship quality among families through egg donation, sperm donor insemination, or natural conception over a 10-year period (14). The study did not show any differences on marital stability between these family groups; only a small proportion of couples with children had separated, and the quality of the relationship of the rest was reported to be good (14). Moreover, a register-based national cohort study conducted in Denmark revealed that undergoing ART treatment did not inherently increase the risk of separation among heterosexual couples over a span of 16 years (15). Furthermore, the authors illustrated that having joint children acted as a protective factor, whereas childlessness significantly elevated the risk of relationship dissolution (15). On the contrary, a study conducted in Sweden by Sydsjö et al. demonstrated that the presence of children did not significantly impact marital stability or quality (10, 11).

To date, studies examining the link among relationship stability, type of infertility treatment (with or without donated gametes), and treatment success have been limited and inconsistent. Furthermore, the majority of them included only heterosexual couples, lacked a prospective design, did not include both partners in the couple, and did not follow study participants over the long term. Therefore, the current study aims primarily to explore whether the type of infertility treatment (with or without donated gametes), sexual orientation of the couples, and the birth of a child are associated with an increased risk of separation among couples undergoing assisted reproduction. Additionally, it aims to investigate separation experiences, custody arrangements, and relationship satisfaction at the time of treatment.

The following specific research questions were examined:

1. Does the separation rate 8–10 years after assisted reproduction differ among: (a) couples treated with donated compared to autologous gametes? (b) lesbian compared to heterosexual couples? (c) couples with child(ren) after ART compared to those without?
2. Do separation experience and custody arrangements differ among separated individuals treated with various types of ART?
3. Is the level of relationship satisfaction within couples at time of treatment associated with separation risk 8–10 years after treatment?

## Material and methods

### Study design

The current study is a prospective multicenter cohort study, conducted as part of the ‘Swedish Study on Gamete Donation’ (SSGD), which is a longitudinal project aimed at exploring various psychosocial aspects among gamete donors, gamete recipients, as well as IVF controls (i.e. heterosexual couples treated with their own gametes). Couples approved for gamete donation treatment between 2005 and 2008 at one of Sweden’s seven university hospitals (located in Stockholm, Gothenburg, Uppsala, Umeå, Linköping, Örebro, and Malmö) were consecutively approached and invited to participate in this study. Furthermore, all heterosexual couples undergoing treatment with autologous gametes (IVF-controls) at the University Hospitals in Gothenburg, Uppsala, Umeå, Linköping, and Örebro in the same time period were also invited to participate. All individuals included in this study received publicly subsidized treatment. Assisted reproduction with donated gametes to single women and embryo-/double donation were not permitted in Sweden at the time. According to Swedish legislation, all individuals eligible for ART treatment with donated gametes underwent compulsory medical and psychosocial evaluation conducted by a fertility specialist and a behavioral expert (16). This evaluation aimed to assess parenting adequacy and ensure that the child would grow under good circumstances. Although corresponding legal requirements do not exist for couples treated with their own gametes, prior reports indicate that fertility specialists require candidates to meet similar standards before accepting them for treatment (16). Furthermore, all individuals undergoing publicly subsidized treatment in Sweden (regardless of whether donated gametes were used) have to meet additional eligibility criteria regarding age, body mass index (BMI), as well as stable social and financial circumstances. All individuals treated were required to be in a committed cohabiting relationship (registered partnership/marriage). People who did not speak and/or read Swedish or did not complete one round of ART treatment were excluded from study participation.

In the SSGD, data collection for couples undergoing ART has been conducted in four waves, irrespective of treatment outcome. Participants completed individual postal questionnaires at treatment start (T1), 2 months after treatment (T2), around 3 years after treatment (T3), and 8–10 years after treatment (T4). A fifth wave of data collection (T5) was conducted in 2022–2023 with only those couples who had adolescent children via ART, that is excluding those who had not conceived a child 2005–2010. The questions included at the surveys (T1–T5) varied. Separation status was only inquired about during the fourth and fifth waves of data collection (T4 and T5). The present study was based on the fourth wave of data collection (T4), as having children (or not) was one factor under investigation.

### Study population

The study exposure regarded the origin and the type of gametes utilized in the fertility treatment (donated vs. non-donated/autologous, oocytes vs. sperm) along with the sexual orientation of the treated individuals (heterosexuals/lesbians). Consequently, the study population comprised four groups: heterosexual couples undergoing fertility treatment with either donated oocytes (Oocyte Donation, OD), donated sperm (Heterosexual Sperm Donation, HtSD), or autologous gametes (IVF), as well as lesbian couples undergoing fertility treatment with donated sperm (Lesbian Sperm Donation, LeSD). Treatment with donated sperm was carried out either through donor inseminations or IVF/ICSI with donated sperm, while treatment with donated oocytes or autologous gametes involved the use of IVF/ICSI. Out of the 1,196 individuals who were included as study participants in the SSGD and completed the baseline questionnaire (T1) (302 IVF, 330 LeSD, 307 OD, and 257 HtSD), a total of 660 (55.2%) individuals participated in the present follow-up survey (8–10 years after initiation of treatment) (participation rate 52.3% IVF, 61.8% LeSD, 50.2% OD, and 56.0% HtSD). The study questionnaire and participant information sheet for this follow-up were mailed to all individuals who participated at T1, along with a stamped reply envelope. Two reminders were sent if there was no response.

### Study data

The follow-up questionnaire completed 8–10 years after ART treatment initiation (T4) included inquiries about demographic data such as the age of the treated woman/co-parent (years), gender (male and female), the role of the spouse in treatment (treated woman/co-parent), occupation (full time/part time employment, student/disability pension/job seeker, and other), education (≤12 years, > 12 years), the presence of biological/adoptive/step children prior to treatment (yes/no), chronic diseases (yes/no), perceived current health status (very good/good, fair, and bad/very bad), and whether the ART treatment resulted in live born children (yes/no). The primary study outcome focused on separation status of the participants (separated/non-separated). Secondary outcomes included separation-related variables assessed only among separated individuals. These questions addressed whether the separation caused the participant physical or mental suffering, reinforced feeling of loneliness *versus* freedom, or improved individual well-being. Participants were also asked about their relationship with their former partner and whether they had entered into new relationships. For separated individuals with children, additional questions covered family counseling, mediation talks, the form of custody, custody disputes, and personal experiences with the custody arrangement.

To assess the perceived relationship satisfaction among individuals/couples at the beginning of the study (T1) in relation to their separation status 8–10 years later (T4), we utilized the Swedish version of the ENRICH inventory (Evaluating and Nurturing Relationship Issues, Communication and Happiness), an inventory originally developed by Olsson and colleagues (17). The ENRICH is widely marketed, particularly in the United States, as the primary evidence-based premarital and marriage assessment tool, capable of predicting divorce with 85% accuracy (18). The primary aim of the inventory is to aid couples get insights aimed at strengthening their relationship and thereby reducing the risk of separation. A detailed description of the ENRICH is provided in the publication by Borneskog et al. (13) arising from the same initial population. However, the study by Borneskog et al. included only two treatment groups compared to four in the present one. In summary, this inventory has been demonstrated to provide a valid and reliable assessment of an individual’s perceived marital/partner satisfaction within their current relationship across the following 10 dimensions/subscales: Personal issues, Communication, Conflict resolution, Financial management, Leisure activities, Sexual relationship, Children and parenting, Family and friends, Egalitarian roles, and Conception of life. Subscale scores range from 10 to 50 with a higher score indicating greater perceived relationship. The ENRICH inventory was completed by all participating individuals at T1, and there were no missing data. To enhance the interpretation of the findings and improve practical usability, we decided to dichotomize the scores in the inventory. This decision was based on the assumption that individuals with high relationship satisfaction, as indicated by their high scores, had a lower risk of considering separation compared to individuals already dissatisfied at treatment commencement. The total ENRICH score, ranging from 100 to 500, was dichotomized into high and low categories, with scores of 400 and above considered high and scores below 400 coded as low. Similarly, each of the 10 ENRICH subscale scores was dichotomized with values of 40 or above classified as high scores and values below 40 classified as low. These cutoffs correspond to the 20^th^ percentile (i.e. 20% scored below the cut-off and 80% scored above the cut-off). Furthermore, in order to pair the (different) ENRICH scores within the couple, the scores were dichotomized into (1) both or one of the persons within the couple scored low and (2) both persons within the couple scored high.

### Statistical analysis

Data processing in this study was performed with the Statistics Package for the Social Science (SPSS) version 28.0 (IBM Inc., Armonk, NY, USA). Questions where both participating spouses were expected to provide unanimous answers, such as separation status, family counseling, mediation talks, custody disputes, as well as custody arrangements, were analyzed at the couple level. If only one spouse participated in this follow-up study, their response was considered representative of the couple. All other questions were analyzed at the individual level. Categorical variables in research questions 1 and 2 were analyzed with the Pearsons’s Chi square and Fisher’s exact test and are presented as number (*n*) and percent (%), while continuous data were compared with the use of Mann–Whitney U/Wilcoxon test and presented as median, minimum, and maximum values. Research question 1 was assessed in the entire study population, whereas question 2 was specifically examined among separated individuals. Both questions were investigated in relation to treatment group.

The relationship between the ENRICH scores of both spouses at treatment commencement was investigated including both total scores and the 10 subscales. This analysis aimed to determine whether both spouses reported high scores (indicating a high degree of relationship satisfaction for both) or if at least one reported a low score (indicating a low degree of relationship satisfaction for at least one spouse). The spousal paired ENRICH scores were compared across different treatment groups and explored in relation to separation status using the Chi-square test. Furthermore, multivariable logistic regression models were utilized to estimate the odds ratio for separation 8–10 years after ART commencement, as outlined in research question 3. In those analyses, the paired ENRICH scores of the spouses at treatment start and the presence of ART-conceived children after treatment were included in the models. Each subscale and total scale were modeled separately. Also, to investigate potential group differences, data were stratified by type of treatment, and the same models were applied as for the total population. The included independent variables were identified by reviewing existing literature. The logistic regression models are presented as odds ratios (ORs), and 95% confidence intervals (CI) were calculated. A significance level below 5% (*P* < 0.05) was employed. Given that this study constitutes a follow-up of individuals recruited at a prior time-point, with different primary outcomes, no sample size calculation was performed a priori.

### Ethics approval

Approval was obtained from the Regional Ethics Review Board in Linköping (Ethics approval numbers M29-05, M29-05/1-06, and 2013/299-31). The procedures used in this study adhere to the tenets of the Declaration of Helsinki.

## Results

### Demographic data

Demographic data are presented in Table 1. In total, 660 individuals participated in this follow-up study, comprising 206 (31.2%) males and 454 (68.8%) females, representing 378 different couples. The proportion of couples where both partners responded to the survey was similar across the treated groups (84.4% OD, 81.9% HtSD, 82.8% LeSD, and 86.7% IVF) (*P* > 0.05).

**Table 1:** Demographic data of study participants in relation to treatment group (*N* = 660).

Baseline characteristics	Categories	OD (*N* = 154) *n* (%)	HtSD (*N* = 144) *n* (%)	LeSD (*N* = 204) *n* (%)	IVF (*N* = 158) *n* (%)	*P*
Gender	Female	84 (54.5)	80 (55.6)	204 (100)	86 (68.8)	<0.001
Age (Median, Min-Max)	Treated woman	43 (35–51)	42 (32–49)	42 (29–49)	41 (32–51)	0.532
	Female/Male Co-parent	44.5 (37–59)	44 (32–61)	42 (32–57)	43.5 (31–64)	
Education	>12 years	85 (55.6)	79 (54.9)	131 (64.2)	88 (56.4)	0.227
Occupation	Full time employment	124 (81.6)	102 (71.3)	149 (74.5)	108 (68.8)	0.262
	Part time employment	24 (15.8)	29 (20.3)	40 (20.0)	40 (25.5)	
	Student/Disability pension/Job seeker	3 (2.0)	6 (4.2)	7 (3.5)	4 (2.5)	
	Other	1 (0.7)	6 (4.2)	5 (2.0)	5 (3.2)	
Health status	Very good/good	130 (86.1)	122 (85.3)	175 (87.5)	136 (86.6)	0.639
	Fair	19 (12.6)	20 (14.0)	25 (12.5)	18 (11.5)	
	Bad/Very bad	2 (1.3)	1 (0.7)	0 (0.0)	3 (1.9)	
Chronic diseases	Yes	32 (21.2)	46 (32.4)	52 (26.0)	43 (27.2)	0.189
Biological/adoptive/step children prior to ART treatment	Yes	19 (12.3)	13 (9.0)	34 (16.7)	19 (12.0)	0.199
Children after ART treatment	Yes	94 (61.0)	113 (78.5)	150 (73.5)	87 (55.1)	<0.001

OD, oocyte donation; HtSD, heterosexual couple with sperm donation; LeSD, lesbian couple with sperm donation; IVF, *in vitro* fertilization with autologous gametes.

No differences were detected between the four treatment groups regarding the age of the treated woman, the education level, occupation status, perceived health status, occurrence of chronic diseases at the time of follow-up, or the age of the co-parent (*P* > 0.05) (Table 1). Although the proportion of individuals with biological/adoptive/step-children prior to ART treatment did not differ among treatment groups, a higher proportion of individuals having children after ART was observed among those treated with sperm donation compared to the other groups (*P <* 0.001) (Table 1).

### Separation status

In this follow-up study, a significantly higher rate of separation was reported among lesbian couples undergoing sperm donation compared to the other treatment groups. At the couple level, the separation rate, for the LeSD group, was 38.5% contrasted with 11.1% for IVF couples, 15.7% for heterosexual couples treated with donated sperm, and 17.0% for heterosexual couples treated with donated oocytes (*P <* 0.001). Similar findings were observed when examining separation status in relation to the sexual orientation of the couples (heterosexual vs. lesbian couples), with lesbian couples exhibiting an almost threefold higher rate of separation compared to heterosexual individuals (38.5% vs. 14.6%, respectively) and a twofold higher rate for couples treated with donated as opposed to autologous gametes (25.3% vs. 11.1%, respectively) (Table 2). No other differences were noted regarding demographics and separation status (Table 3). Separated individuals perceived their health status to be equally good compared to non-separated individuals. The proportion of children (prior to or after ART treatment) was similar between separated and non-separated individuals (Table 3).

**Table 2:** Treatment group in relation to separation status (couple level).

Treatment aspects	Categories of treatment aspects	Separated couples (*N* = 83)	Non-separated couples (*N* = 295)	*P*
Sexual orientation	Heterosexual	38 (14.6)	223 (85.4)	<0.001
	Homosexual	45 (38.5)	72 (61.5)	
Gamete origin	Donated	73 (25.3)	215 (75.7)	0.004
	Autologous	10 (11.1)	80 (88.9)	
Treatment group	OD	15 (17.0)	73 (83.0)	<0.001
	HtSD	13 (15.7)	70 (84.3)	
	LeSD	45 (38.5)	72 (61.5)	
	IVF	10 (11.1)	80 (88.9)	

**Table 3:** Demographic data of study participants in relation to separation status (individual level).

Baseline characteristics	Categories	Separated individuals (*N* = 122)	Non-separated individuals (*N* = 532)	*P*
		*n* (%)	*n* (%)	
Gender	Male	20 (16.4)	185 (34.8)	<0.001
Biological/adoptive/step children prior to ART treatment	Yes	18 (14.8)	66 (12.4)	0.484
Occupation	Full time employment	98 (80.3)	384 (72.6)	0.270
	Part time employment	17 (13.9)	116 (21.9)	
	Student/Disability pension/Job seeker	4 (3.3)	16 (3.0)	
	Other	3 (2.5)	13 (2.5)	
Education	>12 years	71 (58.2)	308 (58.2)	0.996
Health status	Very good/good	98 (80.3)	464 (87.9)	0.083
	Fair	22 (18.0)	60 (11.4)	
	Bad/very bad	2 (1.6)	4 (0.8)	
Chronic diseases	Yes	30 (24.8)	143 (27.0)	0.615
Children after ART	Yes	40 (32.8)	175 (32.9)	0.982
Number of respondents per couple	Two/couple	78 (63.9)	473 (88.9)	<0.001

OD, oocyte donation; HtSD, heterosexual couple with sperm donation; LeSD, lesbian couple with sperm donation; IVF, *in vitro* fertilization with autologous gametes.

### Separation aspects and custodian arrangements after separation

The results of the subgroup analysis focusing solely on separated individuals at T4 are presented in Table 4. A higher, albeit not statistically significant, proportion of lesbian participants reported having a new partner at follow-up compared to individuals in other treatment groups (47.7% vs. 21.1–31.6%). Furthermore, family counseling and relationship mediation were more frequently reported by couples treated with donated gametes compared to couples treated with autologous gametes [(37/71 vs. 0/10) and (11/61 vs. 0/10), respectively] (Table 4). In addition, couples undergoing treatment with donated sperm (i.e. LeSD and HtSD) reported a higher frequency of having children through ART compared to other treatment modalities (approximately 70% compared to 40%, *P* = 0.073), with the association approaching statistical significance. Finally, no differences were observed among the four treatment groups regarding self-reported physical or psychological suffering due to separation, feelings of loneliness or freedom, improved well-being, or improved relationship with the former partner (Table 4).

**Table 4:** Separation-related variables in relation to treatment group [analysis performed only among separated individuals (*n* = 122)].

Separation-related variables	Response	OD (*N* = 20) *n* (%)	HtSD (*N* = 19) *n* (%)	LeSD (*N* = 69) *n* (%)	IVF (*N* = 14) *n* (%)	*P*
New partner (male or female)	Yes	4 (21.1)	6 (31.6)	31 (47.7)	4 (30.8)	0.141
Children after ART treatment^1^	Yes	6 (40.0)	9 (69.2)	32 (71.1)	4 (40.0)	0.073
Family counseling with ex-partner^1^	Yes	8 (53.3)	8 (61.5)	21 (48.8)	0 (0.0)	0.017
Relationship mediation with ex-partner^1^	Yes	2 (13.3)	5 (38.5)	4 (9.1)	0 (0.0)	0.026
Separation caused physical suffering^2^	Agree	4 (21.1)	3 (15.8)	7 (10.1)	0 (0.0)	0.176
	Neutral	2 (10.5)	0 (0.0)	4 (5.8)	0 (0.0)	
	Disagree	13 (68.4)	15 (78.9)	58 (84.1)	11 (91.7)	
	No opinion	0 (0.0)	1 (5.3)	0 (0.0)	1 (8.3)	
Separation caused psychological suffering^sup>2^	Agree	15 (75.0)	8 (42.1)	27 (39.7)	4 (33.3)	0.277
	Neutral	0 (0.0)	2 (10.5)	3 (4.4)	1 (8.3)	
	Disagree	5 (25.0)	9 (47.4)	37 (54.4)	7 (58.3)	
	No opinion	0 (0.0)	0 (0.0)	1 (1.5)	0 (0.0)	
Feeling of loneliness after separation^2^	Agree	9 (45.0)	4 (21.1)	9 (13.0)	2 (16.7)	0.104
	Neutral	1 (5.0)	1 (5.3)	3 (4.3)	1 (8.3)	
	Disagree	10 (50.0)	14 (73.7)	57 (82.6)	9 (75.0)	
Sense of freedom after separation^2^	Agree	11 (55.0)	10 (52.6)	48 (69.6)	6 (50.0)	0.066
	Neutral	0 (0.0)	4 (21.1)	5 (7.2)	4 (33.3)	
	Disagree	8 (40.0)	5 (26.3)	15 (21.7)	2 (16.7)	
	No opinion	1 (5.0)	0 (0.0)	1 (1.4)	0 (0.0)	
Improved relationship with ex-partner after separation^2^	Agree	5 (25.0)	4 (21.1)	25 (36.8)	3 (25.0)	0.445
	Neutral	2 (10.0)	7 (36.8)	13 (19.1)	3 (50.0)	
	Disagree	12 (60.0)	8 (42.1)	26 (38.2)	6 (25.0)	
	No opinion	1 (5.0)	0 (0.0)	4 (5.9)	0 (0.0)	
Improved well-being after separation^2^	Agree	11 (55.0)	13 (68.4)	48 (70.6)	10 (76.9)	0.234
	Neutral	1 (5.0)	3 (15.8)	12 (17.6)	2 (15.4)	
	Disagree	7 (35.0)	3 (15.8)	6 (8.8)	1 (7.7)	
	No opinion	1 (5.0)	0 (0.0)	2 (2.9)	0 (0.0)	

OD, oocyte donation; HtSD, heterosexual couple with sperm donation; LeSD, lesbian couple with sperm donation; IVF, in vitro fertilization with autologous gametes.

1 The rates are calculated at the couple level.

2 The rates are calculated at the individual level.

A higher rate of joint shared child custody (i.e. alternating week schedules) as opposed to other forms of custodial arrangements (such as every 2 weekends, sole custody, or other) was reported by LeSD separated individuals compared to separated individuals from other treatment groups (25/30 vs. 9/15); however, the results did not reach statistical significance (*P* > 0.05). The vast majority of study participants (>75%) did not report any custody disputes and were overall satisfied with the custody distribution, with 86–90% of respondents rating the distribution as very good/good; no differences were observed between the four treatment groups (data not shown).

### ENRICH scores and prediction models

Comparison of separated and non-separated couples at T4 showed significantly more dissatisfaction with relationship quality (in at least one of the spouses) among separated OD couples, and this concerned the total ENRICH score, as well as the subscales Communication, Financial management, Sexual relationship, Family and Friends, and Egalitarian roles (Table 5). Separated couples in the LeSD group displayed a higher level of dissatisfaction than their non-separated counterparts in the total score but not in the subscales. Conversely, the IVF group exhibited discordance solely on the Egalitarian roles’ subscale, while the HtSD group showed no group differences between separated and non-separated couples, neither for the total ENRICH score nor the subscales.

**Table 5:** ENRICH spousal paired scores at treatment start stratified by treatment group (couple level).

ENRICH domain	OD			HtSD			LeSD			IVF			Total		
	Separated couples (*N* = 15) *n* (%)	Non-separated couples (*N* = 73) *n* (%)	*P* *	Separated couples (*N* = 13) *n* (%)	Non-separated couples (*N* = 70) *n* (%)	*P* *	Separated couples (*N* = 45) *n* (%)	Non-separated couples (*N* = 72) *n* (%)	*P* *	Separated couples (*N* = 10) *n* (%)	Non-separated couples (*N* = 80) *n* (%)	*P* *	Separated couples (*N* = 83) *n* (%)	Non-separated couples (*N* = 295) *n* (%)	*P* *
Total score			0.037			0.095			0.030			0.742**			0.172
Low satisfaction	8 (53.3)	19 (26.0)		7 (53.8)	21 (30.0)		12 (26.7)	8 (11.1)		4 (40.0)	39 (48.8)		31 (37.3)	87 (29.5)	
High satisfaction	7 (46.7)	54 (74.0)		6 (46.2)	49 (70.0)		33 (73.3)	64 (88.9)		6 (60.0)	41 (51.2)		52 (62.7)	208 (70.5)	
Personality			0.141			0.850			0.584			0.821**			0.838
Low satisfaction	7 (46.7)	20 (27.4)		5 (38.5)	25 (35.7)		12 (26.7)	16 (22.2)		4 (40.0)	35 (43.8)		28 (33.7)	96 (32.5)	
High satisfaction	8 (53.3)	53 (72.6)		8 (61.5)	45 (64.3)		33 (73.3)	56 (77.8)		6 (60.0)	45 (56.3)		55 (66.3)	199 (67.5)	
Communication			0.033			0.148			0.648			0.740**			0.725
Low satisfaction	7 (46.7)	15 (20.5)		7 (53.8)	23 (32.9)		9 (20.0)	12 (16.7)		4 (40.0)	40 (50.0)		27 (32.5)	90 (30.5)	
High satisfaction	8 (53.3)	58 (79.5)		6 (46.2)	47 (67.1)		36 (80.0)	60 (83.3)		6 (60.0)	40 (50.0)		56 (67.5)	205 (69.5)	
Conflict			0.214			0.239**			0.611			1.000**			0.630
Low satisfaction	9 (60.0)	31 (42.5)		9 (69.2)	35 (50.0)		19 (42.2)	27 (37.5)		6 (60.0)	51 (63.7)		43 (51.8)	144 (48.8)	
High satisfaction	6 (40.0)	42 (57.5)		4 (30.8)	35 (50.0)		26 (57.8)	45 (62.5)		4 (40.0)	29 (36.3)		40 (48.2)	151 (51.2)	
Financial			0.013			0.120			0.532			0.150**			0.934
Low satisfaction	8 (53.3)	16 (21.9)		7 (53.8)	22 (31.4)		9 (20.0)	18 (25.0)		2 (20.0)	35 (43.8)		26 (31.3)	91 (30.8)	
High satisfaction	7 (46.7)	57 (78.1)		6 (46.2)	48 (68.6)		36 (80.0)	54 (75.0)		8 (80.0)	45 (56.3)		57 (68.7)	204 (69.2)	
Leisure			0.088**			0.135**			0.694			0.466**			0.848
Low satisfaction	12 (80.0)	40 (54.8)		10 (76.9)	37 (52.9)		18 (40.0)	25 (34.7)		6 (60.0)	58 (72.5)		46 (55.4)	160 (54.2)	
High satisfaction	3 (20.0)	33 (45.2)		3 (23.1)	33 (47.1)		27 (60.0)	47 (65.3)		4 (40.0)	22 (27.5)		37 (44.6)	135 (45.8)	
Sexual			0.026			1.000**			0.348			0.346**			0.313
Low satisfaction	5 (33.3)	8 (11.0)		1 (7.7)	6 (8.6)		7 (15.6)	7 (9.7)		0 (0.0)	13 (16.3)		13 (15.7)	34 (11.5)	
High satisfaction	10 (66.7)	65 (89.0)		12 (92.3)	64 (91.4)		38 (84.4)	65 (90.3)		10 (100.0)	67 (83.8)		70 (84.3)	261 (88.5)	
Children			1.000**			0.708**			0.301			0.680**			0.618
Low satisfaction	2 (13.3)	9 (12.3)		3 (23.1)	13 (18.6)		9 (20.0)	9 (12.5)		1 (10.0)	16 (20.0)		15 (18.1)	47 (15.9)	
High satisfaction	13 (86.7)	64 (87.7)		10 (76.9)	57 (81.4)		36 (80.0)	63 (87.5)		9 (90.0)	64 (80.0)		68 (81.9)	248 (84.1)	
Family			0.016			0.288			0.205			1.000**			0.177
Low satisfaction	6 (40.0)	10 (13.7)		5 (38.5)	17 (24.3)		8 (17.8)	7 (9.7)		3 (30.0)	24 (30.0)		22 (26.5)	58 (19.7)	
High satisfaction	9 (60.0)	63 (86.3)		8 (61.5)	53 (75.7)		37 (82.2)	65 (90.3)		7 (70.0)	56 (70.0)		61 (73.5)	237 (80.3)	
Egalitarian			0.045**			0.502			0.466			0.012**			0.127
Low satisfaction	12 (80.0)	36 (49.3)		5 (38.5)	34 (48.6)		12 (26.7)	15 (20.8)		2 (20.0)	53 (66.3)		31 (37.3)	138 (46.8)	
High satisfaction	3 (20.0)	37 (50.7)		8 (61.5)	36 (51.4)		33 (73.3)	57 (79.2)		8 (80.0)	27 (33.8)		52 (62.7)	157 (53.2)	
Conception			0.558**			0.629			0.295			0.720**			0.223
Low satisfaction	11 (73.3)	45 (61.6)		8 (61.5)	38 (54.3)		30 (66.7)	41 (56.9)		8 (80.0)	57 (71.3)		57 (68.7)	181 (61.4)	
High satisfaction	4 (26.7)	28 (38.4)		5 (38.5)	32 (45.7)		15 (33.3)	31 (43.1)		2 (20.0)	23 (28.7)		26 (31.3)	114 (38.6)	

OD, oocyte donation; HtSD, heterosexual couple with sperm donation; LeSD, lesbian couple with sperm donation; IVF, in vitro fertilization with autologous gametes.

* Pearson’s chi-square;

** Fisher’s Exact Test.

Multivariate logistic regression analyses were conducted to investigate the influence of couple relationship satisfaction at treatment initiation (T1) and the presence of children after ART on the risk of being separated 8–10 years after treatment (T4). Analyses including the entire study population (couples in all treatment groups) did not show any significant associations between having (not having) a child after treatment or low/high couple relationship satisfaction and the risk of separation (Table 6). When conducting logistic regression analyses stratified by treatment group, we observed in the lesbian group that couples with overall low relationship satisfaction (in at least one of the spouses) were three times as likely to have separated compared with couples agreeing on high relationship satisfaction (OR 2.90, 95% CI 1.07–7.80) (Table 6). No other associations were observed in this group regarding the ENRICH subscales. In the oocyte donation group, low couple relationship satisfaction in the subscales Financial management, Sexual relationship, and Family and Friends was associated with an almost fourfold increase in separation risk. Among heterosexual couples receiving sperm donation, no association with couple relationship satisfaction and separation was observed. Conversely, low couple satisfaction regarding the Egalitarian roles’ subscale among IVF-treated couples appeared to have a protective effect against future separation (OR 0.13, 95% CI 0.02–0.65) (Table 6).

**Table 6:** Multivariable logistic regression on ENRICH spousal paired score at treatment start (T1) and the risk of separation 8–10 years later for the entire population and stratified by type of treatment, presented by odds ratio (OR) and corresponding 95% confidence intervals (CI).

Separation predictors	Total	Stratified by type of treatment			
		OD	HtSD	LeSD	IVF
	OR (95% CI)	OR (95% CI)	OR (95% CI)	OR (95% CI)	OR (95% CI)
**ENRICH spousal paired score:** Total score					
Low satisfaction	1.39 (0.83–2.33)	2.88 (0.90–9.23)	2.60 (0.77–8.82)	2.90 (1.07–7.80)	0.70 (0.18–2.69)
High satisfaction	*Reference*	*Reference*	*Reference*	*Reference*	*Reference*
**Child after treatment**					
No	1.21 (0.73–2.01)	2.04 (0.63–6.58)	1.38 (0.36–5.32)	1.06 (0.45–2.48)	1.84 (0.48–7.02)
Yes	*Reference*	*Reference*	*Reference*	*Reference*	*Reference*
**ENRICH spousal paired score:** Personality					
Low satisfaction	1.04 (0.62–1.75)	2.30 (0.73–7.31)	1.06 (0.31–3.65)	1.28 (0.54–3.03)	0.86 (0.22–3.31)
High satisfaction	*Reference*	*Reference*	*Reference*	*Reference*	*Reference*
**Child after treatment**					
No	1.26 (0.76–2.08)	2.40 (0.76–7.57)	1.62 (0.43–6.05)	1.14 (0.50–2.62)	1.83 (0.48–7.00)
Yes	*Reference*	*Reference*	*Reference*	*Reference*	*Reference*
**ENRICH spousal paired score:** Communication					
Low satisfaction	1.07 (0.63–1.81)	3.06 (0.94–9.98)	2.29 (0.68–7.67)	1.26 (0.48–3.28)	0.62 (0.16–2.39)
High satisfaction	*Reference*	*Reference*	*Reference*	*Reference*	*Reference*
**Child after treatment**					
No	1.25 (0.75–2.08)	2.12 (0.66–6.80)	1.45 (0.38–5.52)	1.14 (0.50–2.62)	1.94 (0.50–7.50)
Yes	*Reference*	*Reference*	*Reference*	*Reference*	*Reference*
**ENRICH spousal paired score:** Conflict					
Low satisfaction	1.11 (0.68–1.81)	1.98 (0.60–5.98)	2.12 (0.58–7.76)	1.22 (0.57–2.60)	0.92 (0.24–3.57)
High satisfaction	*Reference*	*Reference*	*Reference*	*Reference*	*Reference*
**Child after treatment**					
No	1.26 (0.76–2.09)	2.29 (0.73–7.19)	1.36 (0.35–5.23)	1.13 (0.49–2.60)	1.82 (0.47–6.99)
Yes	*Reference*	*Reference*	*Reference*	*Reference*	*Reference*
**ENRICH spousal paired score:** Financial					
Low satisfaction	1.00 (0.59–1.70)	4.32 (1.32–14.13)	2.42 (0.71–8.24)	0.75 (0.30–1.85)	0.30 (0.06–1.52)
High satisfaction	*Reference*	*Reference*	*Reference*	*Reference*	*Reference*
**Child after treatment**					
No	1.26 (0.76–2.09)	2.61 (0.80–8.57)	1.34 (0.34–5.18)	1.14 (0.50–2.63)	2.02 (0.52–7.89)
Yes	*Reference*	*Reference*	*Reference*	*Reference*	*Reference*
**ENRICH spousal paired score:** Leisure					
Low satisfaction	1.02 (0.62–1.67)	3.11 (0.80–12.10)	2.87 (0.68–12.14)	1.26 (0.58–2.72)	0.54 (0.14–2.14)
High satisfaction	*Reference*	*Reference*	*Reference*	*Reference*	*Reference*
**Child after treatment**					
No	1.26 (0.76–2.09)	2.24 (0.71–7.12)	1.11 (0.28–4.46)	1.08 (0.46–2.50)	1.91 (0.49–7.35)
Yes	*Reference*	*Reference*	*Reference*	*Reference*	*Reference*
**ENRICH spousal paired score:** Sexual					
Low satisfaction	1.38 (0.69–2.78)	3.78 (1.01–14.18)	0.79 (0.08–7.40)	1.69 (0.54–5.23)	NA
High satisfaction	*Reference*	*Reference*	*Reference*	*Reference*	*Reference*
**Child after treatment**					
No	1.23 (0.74–2.05)	2.23(0.70–7.15)	1.66 (0.44–6.26)	1.08 (0.46–2.50)	1.85 (0.48–7.16)
Yes	*Reference*	*Reference*	*Reference*	*Reference*	*Reference*
**ENRICH spousal paired score:** Children					
Low satisfaction	1.14 (0.60–2.17)	0.88 (0.16–4.76)	1.42 (0.34–6.03)	1.75 (0.64–4.81)	0.36 (0.04–3.15)
High satisfaction	*Reference*	*Reference*	*Reference*	*Reference*	*Reference*
**Child after treatment**					
No	1.25 (0.76–2.08)	2.44 (0.77–7.72)	1.70 (0.45–6.42)	1.13 (0.49–2.61)	2.12 (0.54–8.35)
Yes	*Reference*	*Reference*	*Reference*	*Reference*	*Reference*
**ENRICH spousal paired score:** Family					
Low satisfaction	1.45 (0.82–2.55)	4.04 (1.16–14.10)	1.90 (0.54–6.63)	1.99 (0.66–5.96)	0.99 (0.23–4.17)
High satisfaction	*Reference*	*Reference*	*Reference*	*Reference*	*Reference*
**Child after treatment**					
No	1.23 (0.74–2.04)	2.29 (0.71–7.41)	1.57 (0.42–5.87)	1.07 (0.46–2.49)	1.83 (0.48–7.00)
Yes	*Reference*	*Reference*	*Reference*	*Reference*	*Reference*
**ENRICH spousal paired score:** Egalitarian					
Low satisfaction	0.65 (0.39–1.08)	3.61 (0.92–14.17)	0.66 (0.20–2.22)	1.36 (0.56–3.30)	0.13 (0.02–0.65)
High satisfaction	*Reference*	*Reference*	*Reference*	*Reference*	*Reference*
**Child after treatment**					
No	1.34 (0.80–2.24)	1.92 (0.59–6.22)	1.64 (0.44–6.09)	1.08 (0.46–2.52)	1.82 (0.45–7.41)
Yes	*Reference*	*Reference*	*Reference*	*Reference*	*Reference*
**ENRICH spousal paired score:** Conception					
Low satisfaction	1.37 (0.81–2.30)	1.56 (0.44–5.48)	1.37 (0.40–4.63)	1.50 (0.69–3.27)	1.70 (0.33–8.70)
High satisfaction	*Reference*	*Reference*	*Reference*	*Reference*	*Reference*
**Child after treatment**					
No	1.24 (0.75–2.06)	2.31 (0.74–7.24)	1.65 (0.44–6.13)	1.10 (0.48–2.55)	1.89 (0.49–7.27)
Yes	*Reference*	*Reference*	*Reference*	*Reference*	*Reference*

Note: Outcome is couple being separated (yes/no, where no is the reference level), adjusted for ENRICH spousal paired score at T1 and Child after treatment. Each ENRICH scale modeled separately.

NA, could not be estimated; OD, oocyte donation; HtSD, heterosexual couple with sperm donation; LeSD, lesbian couple with sperm donation; IVF, in vitro fertilization with autologous gametes; ENRICH, Evaluating and Nurturing Relationship Issues, Communication and Happiness.

## Discussion

### Main findings

In our study, we observed a higher rate of separation among lesbian couples undergoing sperm donation treatment compared to heterosexual couples treated with donated sperm, donated oocytes, or autologous gametes. However, separation risk was associated neither with ART treatment success (i.e. treatment resulting in the birth of a child) or parenthood nor with the occupation or education level of the spouses. Instead, we found that overall relationship dissatisfaction of at least one spouse at the beginning of treatment, as determined by the ENRICH inventory, increased the future risk of separation threefold among lesbian couples. In addition, individuals undergoing oocyte donation treatment who were dissatisfied with their relationship faced a significantly higher risk of separation in the long run. This association persisted even after adjusting for the presence of children after treatment. Consequently, our findings suggest that while separation is more common among lesbian couples, it is among the couples undergoing oocyte donation that the ENRICH inventory can help to predict the risk of separation. This study marks the first prospective examination of intended parents through ART regarding the risk of separation in connection with relationship satisfaction and the type of ART treatment. Considering that donation treatment in Sweden requires a stable relationship at the outset of treatment, as assessed by a behavioral expert, our findings are particularly intriguing. They suggest that an increased risk of separation could potentially be identified before treatment and thus prevented.

One focus of the current study was to assess the impact of assisted reproduction on relationship status among heterosexual and lesbian couples. This exploration aids in understanding how fertility treatment affects diverse family structures and relationship dynamics while promoting inclusivity in reproductive healthcare. Our findings align with previous family demographics in same-sex (female) marriages, where data from other Nordic and Western countries support an almost threefold risk of relationship breakup among lesbian couples compared to heterosexual couples (19). The reasons behind this observation remain unclear, but various relationship dynamics, including heteronormativity and social stress, may play a role. Additionally, gender differences may contribute to these findings, as women are more likely than men to initiate divorce proceedings (20–22). As expected, this would have an impact on the dissolution of heterosexual marriages and, to an even greater extent, lesbian marriages. An unexpected finding was that almost twice as many separated lesbian women had already entered into a new relationship at the time of follow-up compared to separated heterosexual individuals; however, these results did not reach statistical significance.

Although registered partnership was introduced in Sweden in 1995, gender-neutral marriage legislation was adopted in 2009, with both changes overlapping with the time period during which the study was conducted. The contemporary social circumstances could, therefore, have influenced the separation rates estimated in our study. Indeed, in a multi-registry study by Kolk and Anderson conducted in Sweden between 1995 and 2012, assessing opposite and same-sex marriage and divorce, the authors demonstrated an increase in marriage formation associated with the legislation change, which, however, leveled off afterward (23). Although divorce rates in Sweden increased rapidly during the late 1990s and early 2000s, they appear to have stabilized after 2005 when the study was initiated and participants were recruited. Therefore, we can be fairly confident that the legislative and social changes occurring in Sweden at the time did not artificially inflate the estimations of the separation rates calculated in this follow-up study. However, it is important to emphasize that our data on relationship breakup encompass both divorce and dissolution of registered partnership, providing further assurance that our results are not significantly affected.

Finally, a higher proportion of separated couples undergoing donation treatment (HtSD, LeSD, or OD groups) compared to those undergoing autologous treatment (IVF group) reported having utilized family counseling or family mediation. The increased rate of family therapy could be indicative of a greater familiarity and level of comfort among couples undergoing gamete donation in meeting with a therapist, possibly related to the mandatory psychological evaluation they underwent to be eligible for treatment in Sweden.

## Strengths and limitations

This study is one of the largest exploring separation risk among couples treated with either autologous or donated gametes, conducted at a national level with data collected prospectively over a long follow-up period (i.e. 10 years). Furthermore, it includes all assisted reproduction treatment modalities permitted in Sweden during the study period (years 2005–2008). Finally, data collected on the ENRICH inventory were complete, without having any missing values.

This study has, however, some limitations. In 16% of the couples represented in this follow-up, data on separation and separation-related variables are missing from one spouse. Therefore, not all responses on the topic can be validated, potentially affecting estimates at the couple level. However, among the remaining 84% of couples where both spouses responded, there is a good intra-couple agreement on the collected data, reducing the risk of misclassification of our outcome.

We cannot rule out the possibility of selection bias, as a larger proportion of lesbian individuals opted to participate in this follow-up compared to heterosexual individuals (i.e. 61.8% vs. 52.7%), and a higher percentage of them reported being separated. That could potentially result in an overestimation of the separation rates in this treatment group. To address this bias and its impact, separation rates were estimated at the couple rather than the individual level. Additionally, all data were collected from a single country, and all participants underwent publicly funded treatment. Therefore, the findings may not be applicable to couples choosing privately funded cycles, which partly limits the generalizability of our results.

### Clinical implications

By evaluating the relationship satisfaction of couples before undergoing ART and examining the agreement between their responses, healthcare providers can enhance the likelihood of identifying couples with poor relationship quality and a potential risk of separation in the future. Reproductive clinics could, therefore, consider offering couple therapy or other relationship support tools to mitigate this risk and improve the circumstances related to child rearing.

## Conclusion

The presence of children after ART treatment did not impact the separation risk of the treated couples, neither positively nor negatively. A higher proportion of lesbian couples separate within 10 years of treatment with donated sperm compared to heterosexual couples undergoing treatment with donated or autologous gametes. The reason behind this finding remains unclear, and further studies are needed to validate our results and identify the determinants behind it.

## Data Availability

Restrictions apply to the availability of some or all data generated or analyzed during this study to preserve patient confidentiality or because they were used under license. The corresponding author will on request detail the restrictions and any conditions under which access to some data may be provided.
